# Cooperative CO_2_ absorption by amino acid-based ionic liquids with balanced dual sites†

**DOI:** 10.1039/c9ra09293e

**Published:** 2020-02-25

**Authors:** Xiaoyan Chen, Xiaoyan Luo, Jiaran Li, Rongxing Qiu, Jinqing Lin

**Affiliations:** College of Materials Science and Engineering, Huaqiao University Xiamen Fujian 361021 P. R. China chenistrylxy@163.com linlab@hqu.edu.cn

## Abstract

In this study, a variety of functionalized ILs with dual sites including amino acid group (AA) and basic anion (R) were synthesized to investigate the suppression and cooperation between the sites in CO_2_ absorption. The basic anions selected in this study with different basicity include sulfonate (Su), carboxylate (Ac), imidazolium (Im), and indolium (Ind). These ILs ([P_66614_]_2_[AA–R]) were applied to CO_2_ absorption. The results present that CO_2_ capacity increases first and then decreases later with the continuous increase in the activity of the anion site. Combined with CO_2_ absorption experiments, IR and NMR spectroscopic analyses and DFT calculation demonstrate that the ability of one site to capture CO_2_ would be suppressed when the activity of another site is much stronger. Thus, the cooperation of dual site-functionalized ILs and high CO_2_ capacity might be achieved through balancing the two sites to be equivalent. Based on this point, [P_66614_]_2_[5Am–iPA] was further synthesized by taking the advantage of the conjugated benzene ring. As expected, [P_66614_]_2_[5Am–iPA] showed capacity as high as 2.38 mol CO_2_ per mol IL at 30 °C and 1 bar without capacity decrease even after 10 times recycling performance of CO_2_ absorption and desorption.

## Abstract 2

Cooperative CO_2_ absorption by anion functionalized ILs with dual sites including amino acid group (AA) and basic anion (R) could be achieved through regulating the relative activation of two sites.

## Graphical abstract

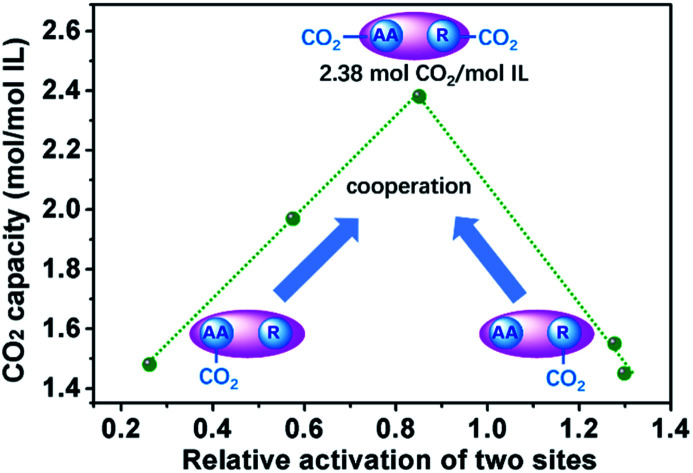

## Introduction

Rapid anthropogenic climate change caused by a large number of greenhouse gases is one of the most significant environmental problems in the world today.^1^ The development of sustainable and environmentally friendly technology to reduce greenhouse gas emissions, particularly carbon dioxide (CO_2_), is the focus of attention in many countries. Unique physicochemical properties of ionic liquids (ILs) such as high thermal stability, low vapor pressure, and tunable properties make ILs suitable for CO_2_ absorption.^2,3^ Functionalized ILs, such as amine,^4,5^ azolium,^6^ phenolate,^7^ and carbene-based ILs^8^ were reported as potential CO_2_ absorption solvents owing to their electron-rich property, or the basicity of ILs. High CO_2_ absorption capacity is one of the performance evaluations of ILs. In this regard, a series of strategies, including tuning the basicity of the active sites,^9–11^ changing the steric hindrance of the ILs,^12,13^ utilization of entropic effects^14,15^ and hydrogen bond formation,^16,17^ were developed to enhance the CO_2_ absorption capacity. However, the CO_2_ capacity of ILs with single site was low compared to those multiple-sites, which have attracted attention to enhance the CO_2_ capacity.

Designing ILs with cooperative sites was considered as attractive to enhance the CO_2_ capacity as well as gas adsorption materials. Vaidhyanathan and Woo^18^ reported CO_2_ capture by an amine-functionalized nanoporous solid with cooperative sites for the low-pressure binding and large uptake of CO_2_. McDonald^19^ reported an energy-saving CO_2_ separation by small temperature or pressure swings *via* the cooperative insertion of CO_2_ in diamine-appended MOFs such as mmen-Mg_2_(dobpdc). It has been found that cooperative CO_2_ capture is a much feasible way to achieve high capacity and reversibility.^20,21^ Recently, cooperative sites were considered in designing ILs. Wang *et al.*^22^ synthesized hydroxyl-pyridinium based ILs with dual cooperative sites to fix CO_2_ for the delocalized π electrons, which enhanced by 85% of the capacity to 1.58 mol mol^−1^ IL. Similarly, the cooperation of Lewis acid–basic reaction and the hydrogen bond interactions of IL with CO_2_ were put to good use to improve CO_2_ capture with imidazolium ILs.^16^ Dai *et al.*^23^ found the dual sites of [P_4442_]_2_[IDA] could be activated by weakening the depression of amine and high capacity of 1.69 mol CO_2_ per mol [P_4442_]_2_[IDA] was achieved. Wang and Cui^24^ synthesized [P_4442_][Suc] with structural preorganization for improving the capture to 1.65 mol mol^−1^ IL of low-concentration CO_2_ as 10 vol% through multiple site cooperation, the capacity would be further enhanced to 2.21 mol mol^−1^ IL at 20 °C and 1.0 bar by tuning the anion substituent group.^25^ However, [apmim]^26^ with the active sites of imidazolium anion and amine group in cation, [aemmim][Tau]^27^ and [aP_4443_][AA]^28^ with an amine group both in cation and anion, [DAIL]^29^ with a dual amine group in cation as well as [Arg], [Lys], [AA–Im]-based ILs^30,31^ with a dual amine group in cation were reported as a CO_2_ absorbent but have capacity just up to equimolar similar to the ILs with a single site, which means one site might be suppressed or inactive. Mu^27^ thought that there is interplay of the dual amine in ILs, which might restrict their ability. As can be seen, cooperation is the key to the high capacity of ILs with dual sites,^32,33^ but the depression of one site is a common problem making the active site to be suppressed and have low CO_2_ capacity.

**Fig. 1:**
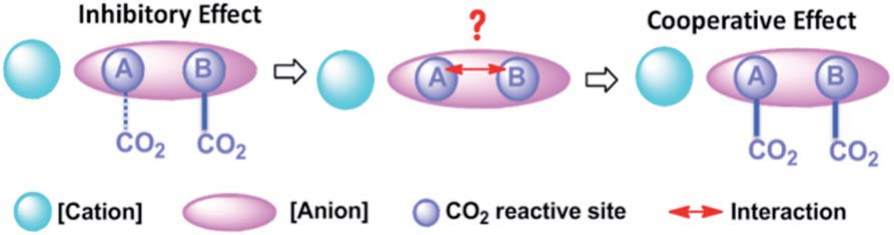
Schematic of the interactions of CO_2_ with dual site-functionalized IL.

**Chart 1:**
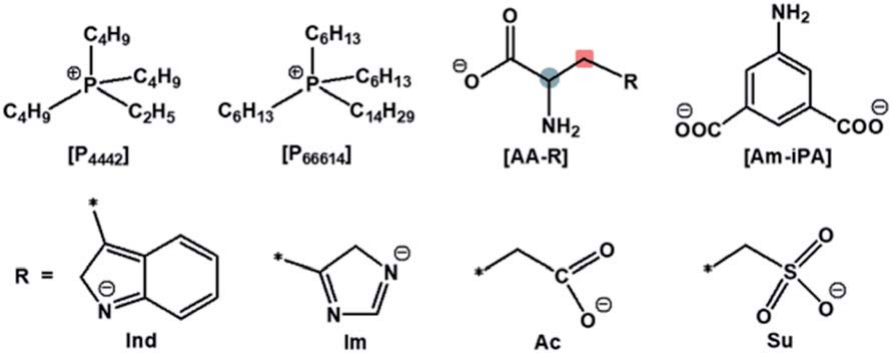
The structure of cations and dual-site anions of ILs.

A dual site-functionalized IL consists of three parts including cation, site A and site B in anion, as shown in Fig. 1. Two possible causes influence the activity of sites, including the interactions between a cation and anion, and the interactions between site A and site B. In our previous study, the effects of cation was investigated and the results indicated that strong interactions between cation and anion would deactivate one site.^34^ In this study, the interplay between dual sites was investigated; amino acids with binary acids are considered as an anion precursor to investigate the depression effects of CO_2_ absorption sites. Phosphonium ions [P_66614_] and [P_4442_] are selected as the cations. The structure of the used ILs is presented in Chart 1. There are two potential sites including amino acid (AA) and anion site (R) in [AA–R]. Thereinto, it has been reported that [P_66614_][AA] could capture CO_2_ efficiently *via* the reaction of an amine group with CO_2_ to carbamic acid. Indolium (Ind) and imidazolium (Im) ions are also good choices for CO_2_ absorption with high capacity, while carboxylate (Ac) anion prefers to react with CO_2_ and should be active to fix CO_2_ efficiently, sulfonate (Su) ion would not react with CO_2_. The results in this study indicate that the dual sites in ILs could cooperate and do their best in the CO_2_ capture if two sites have quite an activity; otherwise, the less active site would be suppressed by another site. Furthermore, [P_66614_]_2_[Am–iPA] was synthesized with equivalent dual sites to cooperative CO_2_ absorption, and the results showed that it presented high capacity as 2.38 mol CO_2_ per mol IL at 30 °C and 1 bar.

## Results and discussion

### Properties of ILs

Some properties of these synthesized ILs such as their thermal property and viscosity were detected, and are shown in Table 1. The decomposition temperature of [P_66614_]_2_[AA–Im] and [P_66614_]_2_[AA–Ind] containing azole groups are 258 °C and 252 °C, respectively. [P_66614_]_2_[Am–PA] with a benzene substituent was stable until the temperature reached above 300 °C, and they are thermally stable enough for the application as a CO_2_ absorbent. The viscosity of these ILs are thousands of cPa and it is considered that the high viscosity was derived from the hydrogen bond formation in amine-based ILs.

**Table 1:** The viscosity and decomposition temperature of typical ILs

Entry	IL	Viscosity^a^ (cPa)	Decomposition temperature^b^ (°C)
1	[P_66614_]_2_[AA–Im]	2693	258
2	[P_66614_]_2_[AA–Su]	4432	296
3	[P_66614_]_2_[AA–Ac]	3076	264
4	[P_66614_]_2_[AA–Ind]	4220	252
5	[P_66614_]_2_[Am–iPA]	5135	320

a Viscosity date were obtained using a Bookfield DV-II+ Pro viscometer at 30 °C.

b Decomposition temperature was measured by DTG with temperature increase from 30 °C to 600 °C at a rate of 10 °C min^−1^ under an argon gas flow.

### CO_2_ absorption

**Fig. 2:**
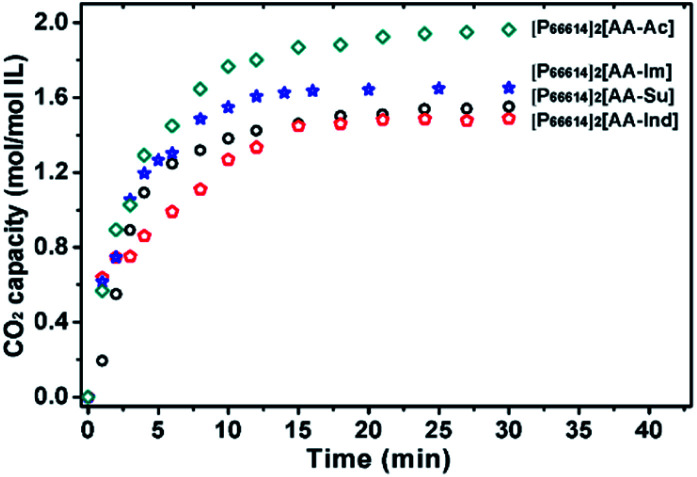
Properties of the CO_2_ absorption of dual site-functionalized ILs at 30 °C and 1 bar CO_2_ pressure.

These ILs were applied to CO_2_ absorption under 1 bar CO_2_ pressure at 30 °C (Fig. 2). [P_66614_]_2_[AA–Ac] showed a high capacity of 1.97 mol CO_2_ per mol IL. With an exchange of a cation, the IL [P_4442_]_2_[AA–Ac] shows a capacity of 2.05 mol CO_2_ per mol IL, with no obvious distinction with the exchange of a phosphonium cation, and this phenomenon is similar to that observed in the previous studies.^34,35^ Thus, the following experiments take [P_66614_] as the ILs' cation. Otherwise, [P_66614_]_2_[AA–Su] presents a CO_2_ capacity of 0.49 mol mol^−1^ IL, which is less than [P_66614_]_2_[AA–Ac] for the sulfonic moiety that does not react with CO_2_. According to the ref. 6, azolium ions such as [Ind] and [Im] prefer to react with CO_2_. [P_66614_]_2_[AA–Ind] and [P_66614_]_2_[AA–Im] with azolium ions were applied to CO_2_ absorption. However, their CO_2_ absorption capacities are 1.45 and 1.55 mol mol^−1^ IL, respectively, which are lower than that of [P_66614_]_2_[AA–Ac]. This means the activity of the CO_2_ absorption site AA or azolium ions might be suppressed.

### The possible mechanism for CO_2_ absorption

**Fig. 3:**
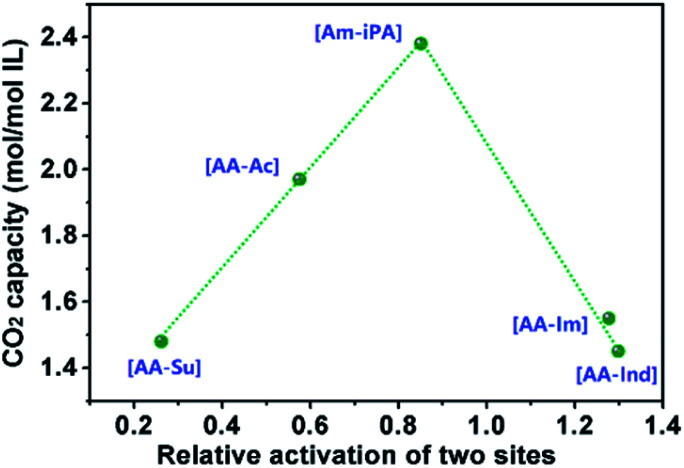
Properties of CO_2_ absorption capacity of dual sites ILs varied with relative activation of two sites presented as Δ*H*(R–CO_2_) divide by Δ*H*(AA–CO_2_).

The possible interaction process controlled by enthalpy was speculated *via* theoretical calculations performed using the Gaussian 03 program at the B3LYP/6-31G++(d,p) level, the optimized structures of the [AA–R], and its CO_2_ complexes are listed in Fig. S1† and the enthalpies are listed in Table 2. It is reported that CO_2_ could be chemisorbed when the reaction enthalpy is less than about −50 kJ mol^−1^.^6^ As seen from Table 2, the amine groups are all active for CO_2_ absorption according to the reaction enthalpy Δ*H*(AA–CO_2_). The reaction enthalpy of CO_2_ with an Ac anion in [AA–Ac] is −61.08 kJ mol^−1^, which means that Ac could react with CO_2_ efficiently, and this result coincides with the absorption capacity of [P_66614_]_2_[AA–Ac] as 1.97 mol CO_2_ per mol IL. The Su anion is not an active site for CO_2_ absorption because the reaction enthalpy of CO_2_ with the Su anion in [AA–Su] is −27.15 kJ mol^−1^, which causes the CO_2_ capacity of [P_66614_]_2_[AA–Su] presenting 0.49 mol mol^−1^ less than [P_66614_]_2_[AA–Ac]. The reaction enthalpies of the amine group and Im anion in [AA–Im] with CO_2_ are −102.28 and −130.68 kJ mol^−1^, which indicates that two sites might react with CO_2_ preferentially. However, [P_66614_]_2_[AA–Im] also showed 0.42 mol CO_2_ per mol IL less than [P_66614_]_2_[AA–Ac] and is similar to [P_66614_]_2_[AA–Ind]. There is an inhibiting effect between sites including anion and amine, where the amine site competes with anion in CO_2_ reaction. The CO_2_ capacity was associated with the relative activation of two sites, which was considered as the value of Δ*H*(R–CO_2_) divided by Δ*H*(AA–CO_2_). The results in Fig. 3 indicate that there is a high CO_2_ capacity when the activation of two sites are almost equivalent, whereas the CO_2_ capacity is much lower.

**Table 2:** Reaction enthalpies of CO_2_ with one site of [AA–R]

[AA–R]	Reaction enthalpy^a^		CO_2_ capacity^b^	Relative activation of two sites^c^
	Δ*H*(AA–CO_2_)	Δ*H*(R–CO_2_)		
[AA–Ac]	−106.15	−61.08	1.97	0.58
[AA–Su]	−103.67	−27.15	1.48	0.26
[AA–Im]	−102.28	−130.68	1.55	1.28
[AA–Ind]	−105.32	−136.78	1.45	1.30
[Am–iPA]	−75.85	−64.53	2.38	0.85

a kJ mol^−1^, the reaction enthalpies were calculated by the Gaussian program at B3LYP/6-31G++(d,p) level.

b mol mol^−1^ IL, CO_2_ absorption was operated at 30 °C, 1 bar.

c Relative activation of two sites was presented as Δ*H*(AA–CO_2_) divide by Δ*H*(R–CO_2_).

**Fig. 4:**
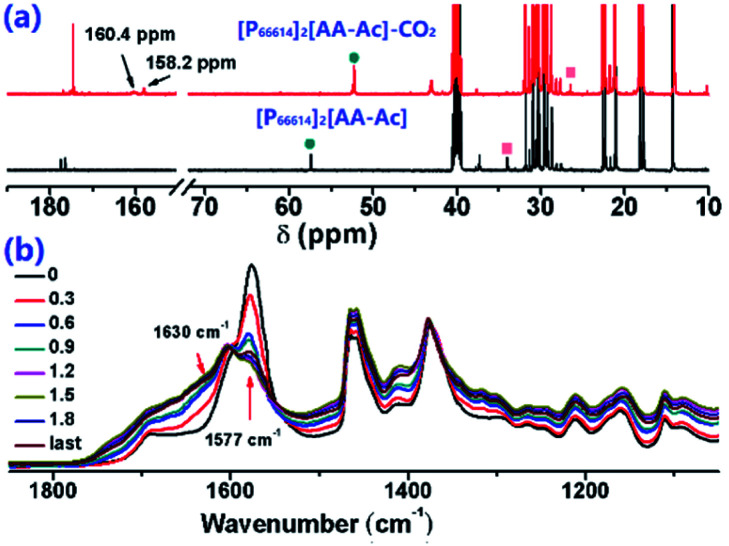
(a) ^13^C NMR spectra of [P_66614_]_2_[AA–Ac] compared with its CO_2_ saturated complex, (b) IR spectra of [P_66614_]_2_[AA–Ac] varied with CO_2_ content.

The CO_2_ absorption with these ILs was investigated *via* IR and ^13^C NMR spectroscopy, as shown in Fig. 4. There are 2 new peaks at 160.4 ppm and 158.2 ppm in the ^13^C NMR spectra compared with CO_2_ saturated [P_66614_]_2_[AA–Ac] and its fresh state, while the chemical shift of the CH (marked with green circle) and CH_2_ (marked with red square) groups have a few ppm changes, which indicates that CO_2_ is fixed in two forms. As seen from the IR spectra of [P_66614_]_2_[AA–Ac] in Fig. 4(b), the vibration absorption of the carboxylate anion at 1585 cm^−1^ shift to 1610 cm^−1^ and the IR absorption intensity of the captured CO_2_ between 1630–1760 cm^−1^ increases with a gradual increase in the CO_2_ content, which indicates that the carboxylate anion assists in CO_2_ absorption.

**Fig. 5:**
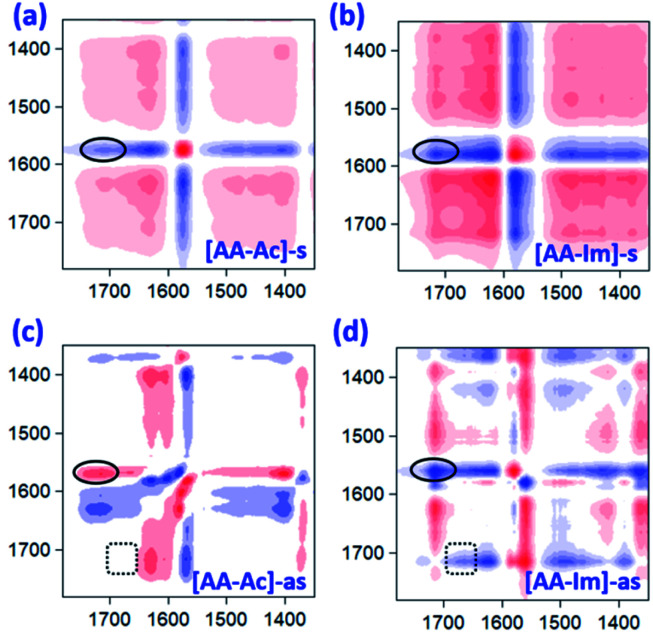
Two-dimensional correction of the IR spectra of typical ILs with CO_2_ content as a variable factor. (a) Synchronous correction of [P_66614_]_2_[AA–Ac]; (b) synchronous correction of [P_66614_]_2_[AA–Im]; (c) asynchronous correction of [P_66614_]_2_[AA–Ac]; (d) asynchronous correction of [P_66614_]_2_[AA–Im]. Red represents positive intensity and blue negative intensity.

**Fig. 6:**
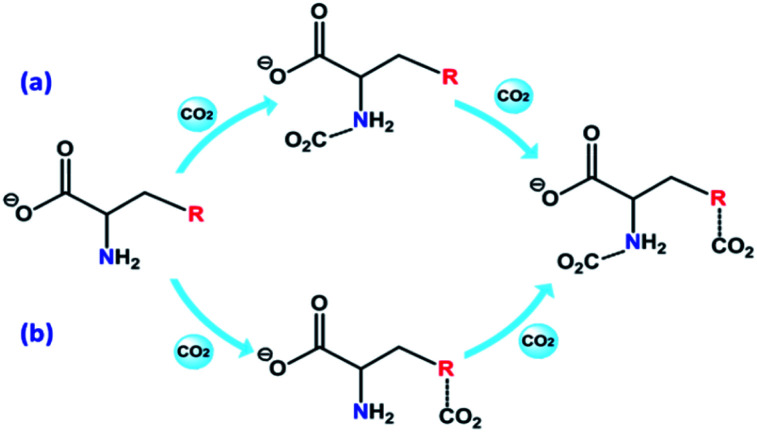
Possible reaction mechanism of dual sites ILs.

The 2D IR spectroscopy is a usual method to study the dynamics of interactions.^36,37^ Thereinto, the IR spectra of ILs associated with the CO_2_ content in 2D correction forms are shown in Fig. 5. Compared with the synchronous and asynchronous correction of [P_66614_]_2_[AA–Ac], it is interesting that the absorption between 1630–1780 cm^−1^ consists of several peaks. There are cross-correlation peaks marked with the elliptical line that appeared at (1715 cm^−1^, 1585 cm^−1^), where 1585 cm^−1^ belongs to the carboxylate anion and 1715 cm^−1^ belongs to the fixed CO_2_. The *Ψ*(1715 cm^−1^, 1585 cm^−1^) in Fig. 5(a) is opposite in signs to *Φ*(1715 cm^−1^, 1585 cm^−1^) in Fig. 5(c), which indicates that the change of 1585 cm^−1^ precedes 1715 cm^−1^. Similarly, with the analysis of the correlation among the peaks of [P_66614_]_2_[AA–Ac] at 1585, 1660 and 1715 cm^−1^, it indicates that the absorption at 1660 cm^−1^ belongs to the fixed CO_2_ with the amine group in [AA–Ac] also follows 1585 cm^−1^, while 1715 cm^−1^ and 1660 cm^−1^ appear simultaneously for no cross-correlation peak at *Φ*(1713 cm^−1^, 1660 cm^−1^), which is marked with dotted square in Fig. 5(c). Thus, the amine site of [AA–Ac] reacts with one CO_2_, then another CO_2_ fixed by an anion site, dual sites of ILs such as [P_66614_]_2_[AA–Ac] could be listed as path (a) in Fig. 6, it presents high capacity of up to 1.97 mol CO_2_ per mol IL owing to the cooperation of amine group and the carboxylate anion.

However, what happens on using [P_66614_]_2_[AA–Im] as a CO_2_ absorption agent?

**Fig. 7:**
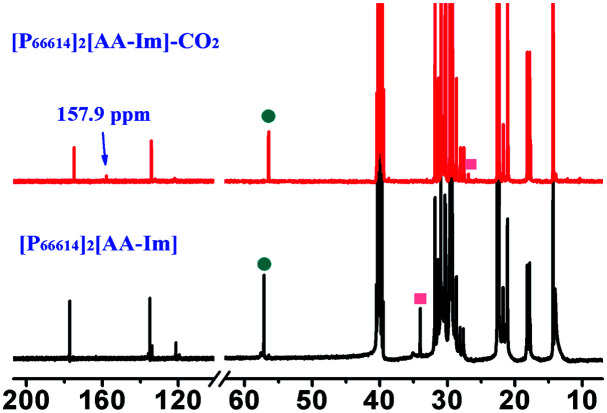
Partial ^13^C NMR spectra of [P_66614_]_2_[AA–Im] compared with [P_66614_]_2_[AA–Im]–CO_2_.

As can be seen from Fig. S3† that the partial IR spectra of [P_66614_]_2_[AA–Im] varied with the CO_2_ content, the IR absorption of the carboxylate anion at 1578 cm^−1^ gets blueshift with the uptake of CO_2_, and the absorption of the fixed CO_2_ with the Im and amine group appear at 1713 and 1664 cm^−1^, respectively. Compared with the synchronous and asynchronous 2D correction IR spectra of [P_66614_]_2_[AA–Im] associated with the CO_2_ content, the sign of *Ψ*(1713 cm^−1^, 1578 cm^−1^) in Fig. 5(b) and *Φ*(1713 cm^−1^, 1578 cm^−1^) in Fig. 5(d) indicate the absorption of fixed CO_2_ by imidazolium at 1713 cm^−1^ before the change in the vibration absorption of the carboxylate anion at 1578 cm^−1^. Similarly, the IR absorption of the fixed CO_2_ with the amine group in [AA–Im] at 1664 cm^−1^ follows 1713 cm^−1^, while the absorption appears at 1664 cm^−1^ prior to 1578 cm^−1^. Thus, the reaction of CO_2_ with dual site ILs could be listed as path (b) in Fig. 6, that the amine site of [AA–Im] reacts with CO_2_ following with CO_2_ fixed by anion site, which is different from [P_66614_]_2_[AA–Ac]. The ^13^C NMR spectra in Fig. 7 shows that one new carbon appeared at 157.9 ppm compared with CO_2_ saturated [P_66614_]_2_[AA–Im] and its fresh state. It is noticed that the chemical shift of CH (marked with a green circle) group shifts tiny, which would be attributed to the reaction of the amine group with CO_2_ be suppressed. A similar phenomenon occurs in CO_2_ absorption with [P_66614_]_2_[AA–Ind]. We thought that cooperative CO_2_ absorption with dual site-functionalized ILs might be liable to occur when the dual sites have the equivalent ability.

### Application of cooperation

**Fig. 8:**
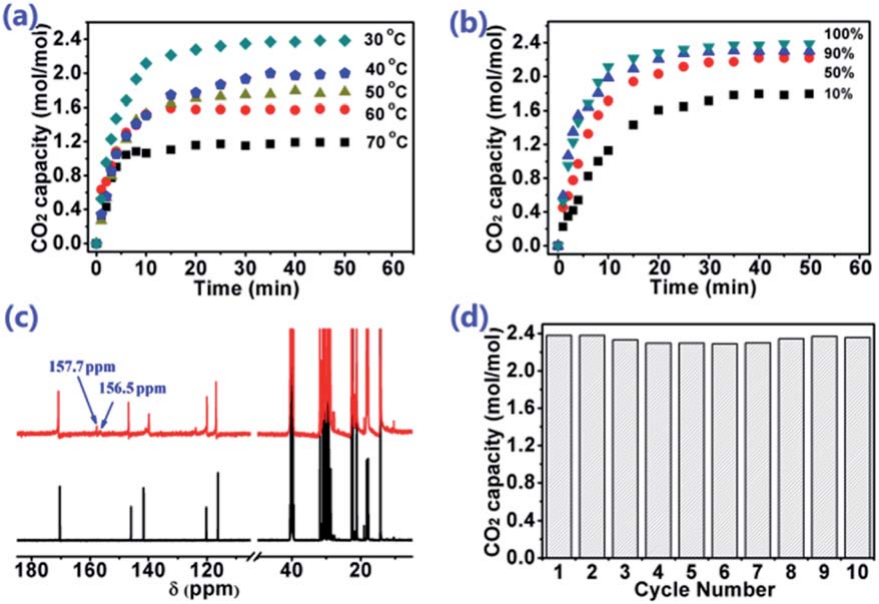
Properties of CO_2_ absorption with [P_66614_]_2_[Am–iPA]. (a) CO_2_ capacity varied with temperature. (b) CO_2_ capacity varied with the CO_2_ content of CO_2_ and N_2_ mixed gas at 1 bar pressure. (c) ^13^C NMR spectra of [P_66614_]_2_[Am–iPA] compared with the [P_66614_]_2_[Am–iPA]–CO_2_ complex. (d) 10 recycle of CO_2_ absorption at 30 °C and 1 bar CO_2_ pressure, and desorption at 80 °C and 1 kPa vacuum for 1 hour.

Based on this opinion that the sites with equivalent ability are more likely to form cooperative CO_2_ absorption, we know that the valence electron delocalizes in the conjugated plane, so that the charge can be dispersed. Thereinto, [P_66614_]_2_[Am–iPA] was synthesized by taking advantage of a benzene ring, which is one of the most common conjugated planes. The reaction enthalpy of CO_2_ with the amine group and carboxylate anion were calculated to predict the possibility of the cooperative CO_2_ absorption of dual sites. The reaction enthalpy of CO_2_ with carboxylate anion and amine in [Am–iPA] are −64.53 and −75.85 kJ mol^−1^, respectively, which indicate that two sites might react with CO_2_, and the reactions are more moderate than with [AA–Im] and [AA–Ind], which is a benefit to CO_2_ desorption. [P_66614_]_2_[Am–iPA] was applied to CO_2_ absorption with capacity as high as 2.38 mol mol^−1^ IL within 20 min at 30 °C. There are two new carbon peaks of captured CO_2_ with [P_66614_]_2_[Am–iPA] that appeared at 157.7 and 156.5 ppm in the ^13^C NMR spectra from Fig. 8(c). In the carbon capture utilization, moisture may be one of the strongest competitors to CO_2_ in the absorption with ILs; therefore, the humid CO_2_ absorption performance with [P_66614_]_2_[Am–iPA] was tested at 30 °C (Fig. S5†). The results indicated that [P_66614_]_2_[Am-iPA] was diluted with 2.5 wt% water, and the CO_2_ absorption capacity of [P_66614_]_2_[Am–iPA] with the copresence of water remained at 8.56 wt%. Compared with the dry CO_2_ absorption capacity of 9.14 wt% (2.38 mol CO_2_ per mol IL), it was demonstrated that a small amount of water in IL did not significantly reduce the CO_2_ capture capability.^33,34^ The influence of temperature and pressure were investigated. The results in Fig. 8(a and b) indicate that the CO_2_ capacity decreases to 1.19 mol mol^−1^ when temperature increase to 70 °C under 1.0 bar, or decreases to 1.79 mol mol^−1^ when the CO_2_ partial pressure decreases to 0.1 bar at 30 °C, which means that the captured CO_2_ could be desorbed by increasing the temperature and decreasing the CO_2_ partial pressure. The thermal stability of [P_66614_]_2_[Am–iPA] was characterized *via* TGA measurement with its decomposition temperature set as 322 °C. Ten consecutive absorption cycles of CO_2_ by [P_66614_]_2_[Am–iPA] are displayed in Fig. 8(d) and exhibit reversibility with the captured CO_2_ being desorbed at 80 °C and 1 kPa vacuum for 1 hour. With the cooperation of dual sites, higher CO_2_ capacity as well as weaker interactions between CO_2_ and IL could be achieved.

## Experimental

### Materials and methods

The used reagents such as dl-aspartic acid (AA–Ac), l-histidine (AA–Im), tryptophan (AA–Ind), dl-homocysteic acid (AA–Su), and 5-aminoisophthalic acid (Am–iPA) were purchased from Aladdin Industrial Co. Tributylethylphosphonium bromide and trihexyl(tetradecyl)phosphonium bromide were purchased from Nanjing Chemlin Biomedical Technology Company. An anion exchange resin in the chloride form (95% purity) and tributylamine (98% purity) were purchased from Sinopharm Group Chemical Company.

### Synthetic procedures

ILs were prepared *via* synthesizing halide ILs, anion exchange, and neutralization with amino acids. Taking the synthesis of trihexyl(tetradecyl)phosphonium tryptophan ([P_66614_]_2_[AA–Ind]) as an example. Trihexyl(tetradecyl)phosphonium bromide ([P_66614_][Br]) was synthesized according to the reported ref. 38. A solution of trihexyl(tetradecyl)phosphonium hydroxide ([P_66614_][OH]) in ethanol was obtained from [P_66614_][Br] by the anion-exchange method,^4^ and equimolar tryptophan was added to the ethanol solution of [P_66614_][OH]; the mixture was dealt with a vacuum-rotary evaporation procedure at 60 °C to remove the most of ethanol after stirring at room temperature for 24 h. Then, all ILs were dried under vacuum at 80 °C for 24 h and freeze-dried to remove possible traces of water.

### Characterization

The synthesized ILs were confirmed *via* NMR and FTIR spectroscopies (see the NMR and IR absorption data in ESI†), and FTIR spectra were recorded using a Nicolet iS50 FT-IR spectrometer. ^1^H and ^13^C NMR spectra were recorded on a 500 MHz Bruker Avance III spectrometer in a deuterated reagent using tetramethylsilane as the standard, and the purities of ILs were calculated based on the NMR spectra. Moreover, the content of the ILs used in this work in higher than 95%. Furthermore, the water content of these ILs was determined by Karl Fischer titration, which was lower than 0.5 wt%. The viscosity of ILs was detected on a Brookfield DV-II+ Pro viscometer at 30 °C. Their thermal stability was analysed *via* TGA on Shimadzu DTG-60H with an increase in temperature from 25 °C to 600 °C with an increased ratio of 10 °C min^−1^ under N_2_ gas protection.

### CO_2_ absorption

CO_2_ absorption experiments with the IL were carried out according to our previous report. CO_2_ desorption was operated under a vacuum pressure of 1 kPa for an hour to recover the IL. CO_2_ capture properties with IL were proposed *via* calculation, and all calculations were performed in this study using the Gaussian 03 programs package. For each set of calculations, we calculated the geometry optimization for the free anion and the cation–CO_2_ complex, and their energies at the B3LYP/6-31G++(d,p) level. 2D correlation IR spectroscopy was acquired *via* a 2D software based on Fourier transform and the analysis was according to the so-called Noda's rule. The intensity of the synchronous 2D correlation spectrum *Φ*(*ν*_1_, *ν*_2_) represents the simultaneous or coincidental changes in two spectral intensity variations measured at *ν*_1_ and *ν*_2_ during the measurement interval of the NH_3_ content, while the intensity of an asynchronous spectrum *Ψ*(*ν*_1_, *ν*_2_) represents sequential or successive but not coincidental changes in spectrum measured separately at *ν*_1_ and *ν*_2_.

## Conclusions

In summary, a variety of dual site-functionalized ILs [P_66614_]_2_[AA–R] were synthesized with an amino acid group and basic anion including sulfonate, carboxylate, imidazolium, and indolium to investigate the suppression and cooperation between each site in CO_2_ absorption. Combined with CO_2_ absorption experiments, IR and NMR spectroscopic analyses, and DFT calculations, CO_2_ absorption results indicated that the CO_2_ capacity increases first but decreases later with the continuous increase in the CO_2_ absorption ability of R. The ability of the amine group to capture CO_2_ would be suppressed when the interactions of another site with CO_2_ is stronger. Thus, the dual site-functionalized ILs might be cooperative to achieve high CO_2_ capacity by balancing two sites to be equivalent. Based on this point, [P_66614_]_2_[Am–iPA] was further synthesized by taking the advantage of a conjugated benzene ring. As expected, [P_66614_]_2_[Am–iPA] showed capacity as high as 2.38 mol CO_2_ per mol IL and without capacity decrease within 10 times recycle performance of CO_2_ absorption and desorption. Cooperation exists widely in a variety of fields as well as gas absorption, and the investigation of the knowledge of suppression would be better to achieve cooperation.

## Conflicts of interest

There are no conflicts to declare.

## Supplementary Material

RA-010-C9RA09293E-s001
