# Supplementary material for: Cooperative CO2 absorption by amino acid-based ionic liquids with balanced dual sites
Source: RSC Adv. 2020 Feb 25;10(13):7751–7. doi: 10.1039/c9ra09293e (PMC9049852; doi:10.1039/c9ra09293e)
Supplement: RA-010-C9RA09293E-s001 [file RA-010-C9RA09293E-s001.pdf]

Electronic Supplementary Material (ESI) for RSC Advances.
This journal is © The Royal Society of Chemistry 2020

Supporting information

Cooperative CO_2 Absorption by Amino Acid based Ionic Liquids with Balanced Dual Sites

Xiaoyan Chen, Xiaoyan Luo*, Jiaran Li, Rongxing Qiu, and Jinqing Lin*

Content:

1. Supporting table..S1
2. Supporting Figures..S3
3. NMR and IR data of amine acid-based ILs..S5
4. References..S7

1. Supporting table

Table S1. Structures, names and abbreviations of ILs discussed in this work.

Structure	Ion name	Abbreviation
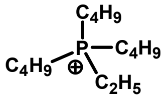	Tributyl(ethyl)phosphonium	[P_{4442}]
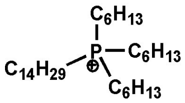	Trihetyl(tetradecyl)phosphonium	[P_{66614}]
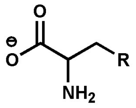	Dual sites ion	[AA-R]
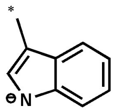	Tryptophan	Ind
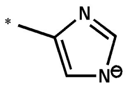	Histidine	Im
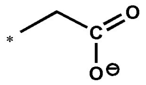	Glutamate	Ac
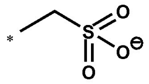	DL-homocysteic Acid	Su
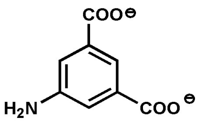	5-Aminoisophthalic Acid	[Am-iPA]

Table S2. The H_2O content in ILs detected by Karl Fisher detector and the ILs' content calculated from NMR spectra.

IL	1st detection (wt.%)	2nd detection (wt.%)	H_2O content (wt.%)	IL content (%)
[P_{66614}]$_2$[AA-Im]	-0.007	-0.005	<0.03	97.1
[P_{66614}]$_2$[AA-su]	-0.002	-0.005	<0.03	97.4
[P_{66614}]$_2$[AA-Ac]	0.403	0.403	0.40	97.5
[P_{66614}]$_2$[AA-Ind]	-0.004	-	<0.03	>99
[P_{66614}]$_2$[Am-iPA]	0.252	-	0.25	97.8

Table S3. Comparation of CO_2 absorption properties of some typical ILs with dual sites.

Absorbent	T (^oC)	CO_2 capacity[a] (mol/mol IL)	reference
$[P_{66614}]_2$[Am-iPA]	30	2.38	This work
$[P_{66614}]_2$[Asp]	30	1.96	1
$[P_{4442}]_2$[IDA]	40	1.69	2
$[P_{4442}]$[Suc]	20	1.87	3
$[P_{4442}]$[Cy-Suc]	20	2.21	4
$[P_{66614}]$[2-Op]	20	1.58	5
$[P_{66614}]$[4-CHO-Im]	30	1.24	6
$[P_{66614}]$[2-Op]	20	1.69	7
[TBP][Arg]	30	1.52 (4.79)[b]	8
[TBP][Lys]	30	1.59 (5.02)[b]	8
1-(2-hydroxyethyl)-2,3-dimethylimidazolium phenoxide	25	1.58	9
$[P_{66614}]$[PhO]	22	0.68[c]	10
$[N_{66614}]$[Lys]	22	2.1	11
$[NH_2$-pmim][Im]	40	~0.75	12

[a] CO_2 capacity was obtained under 1 bar CO_2 pressure. [b] the CO_2 capacity in brackets is belonged to 3% IL aqueous solution. [c] CO_2 capacity was obtained under 1.38 bar CO_2 pressure.

2. Supporting Figures

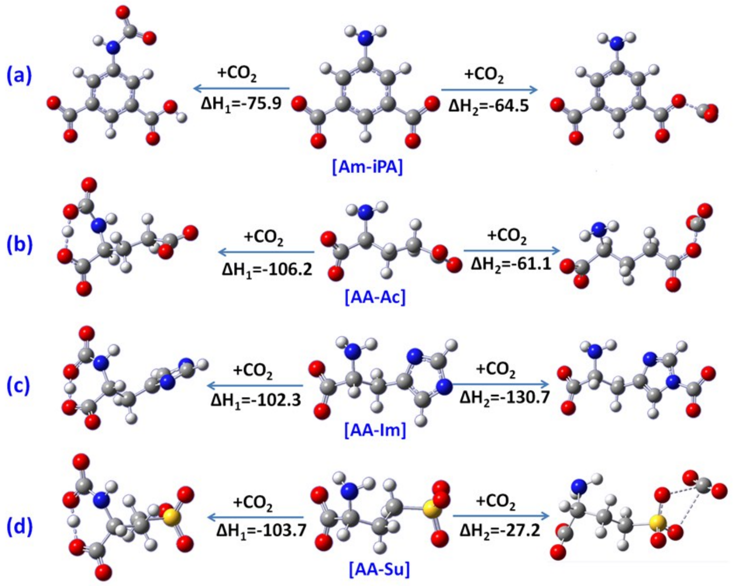

Figure S1. Reaction enthalpies of CO_2 with one site of [AA-anion] calculated by Gaussian program at B3LYP-31++G(d,p) level. ΔH, kJ/mol; gray, C atom; white, H atom; red, O atom; blue, N atom; yellow, S atom.

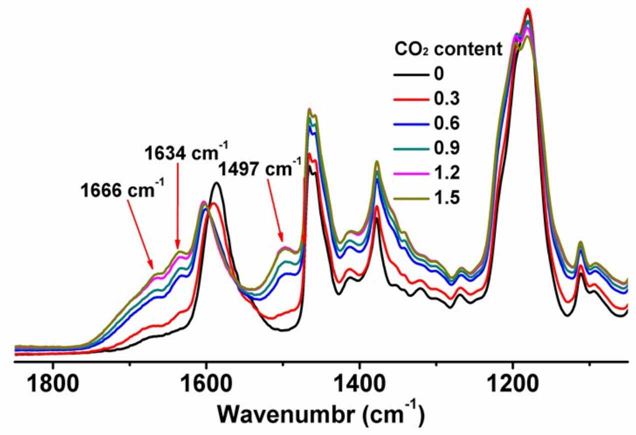

Figure S2. Partial IR spectra of $[P_{66614}]_2$[AA-Su] varied with CO_2 content.

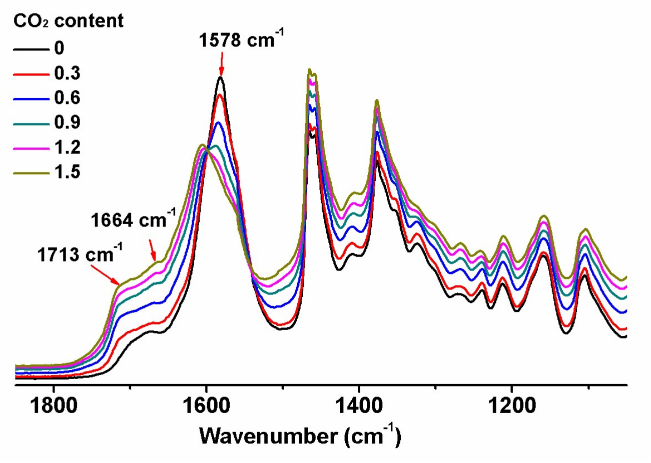

Figure S3. Partial IR spectra of $[P_{66614}]_2$[AA-Im] varied with CO_2 content.

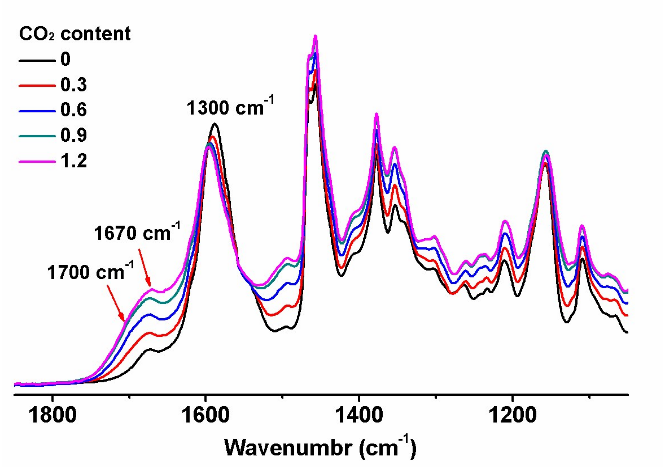

Figure S4. Partial IR spectra of $[P_{66614}]_2$[AA-Ind] varied with CO_2 content.

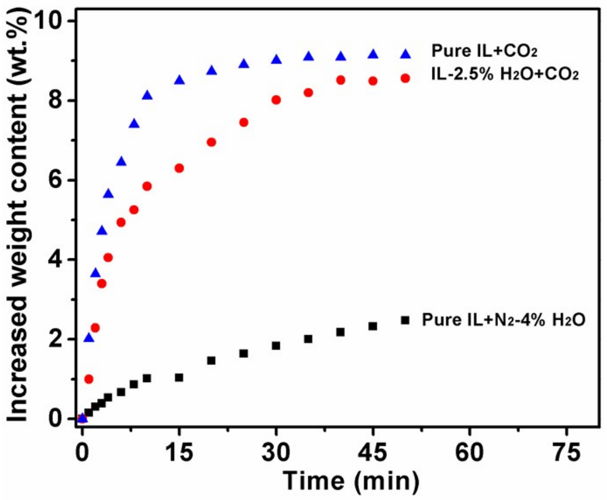

Figure S5. CO_2 and moisture absorption with $[P_{66614}]_2$[Am-iPA] and its H_2O containing complex.

3. NMR and IR data of amine acid-based ILs

$[P_{66614}]_2$[AA-Ac]: ^{1}H NMR (DMSO, ppm): 0.851 (t, 6H,CH_3), 0.882 (t, 18H, CH_3), 1.237 (m, 36H, CH_2), 1.378 (m, 16H, CH_2), 1.448 (m, 16H, CH_2), 1.557 (m, 4H, CH_2), 1.728 (m, 4H, CH_2), 2.181 (m, 16H, CH_2), 2.582 (m, 1H, CH); ^{13}C NMR(DMSO, ppm): 14.231, 17.313, 17.699, 20.647, 21.834, 22.105, 28.181, 28.749, 29.046, 29.106, 29.743, 29.863, 30.453, 31.323, 33.516, 36.826, 57.014, 176.128, 177.002.

$[P_{66614}]_2$[AA-Ac]-CO_2: ^{1}H NMR (DMSO, ppm): 0.857 (t, 6H,CH_3), 0.877 (t, 18H, CH_3), 1.243 (m, 32H, CH_2), 1.285(m, 28H, CH_2), 1.379 (m, 16H, CH_2), 1.460 (m, 16H, CH_2), 1.567 (m, 4H, CH_2), 1.680 (m, 2H, CH_2), 1.979 (m, 1H, CH), 2.091(m, 2H, CH_2), 2.193(m, 16H,CH_2); ^{13}C NMR(DMSO, ppm): 13.559, 17.345, 17.730, 20.661, 21.802, 22.087, 28.225, 28.776, 29.073, 29.711, 29.832, 30.402, 31.337, 37.021, 42.803, 51.717, 157.539, 159.935, 174.052, 176.380.

$[P_{66614}]_2$[AA-Im]: ^{1}H NMR (DMSO, ppm): 0.858 (t, 6H,CH_3), 0.879 (t, 18H, CH_3), 1.243 (m, 32H, CH_2), 1.285(m, 28H, CH_2), 1.378 (m, 16H, CH_2), 1.456 (m, 16H, CH_2), 1.566 (m, 4H, CH_2), 2.191 (m, 12H, CH_2), 2.389 (m, 2H, CH_2), 2.798 (m, 2H, NH_2), 2.931 (t, 2H, CH_2), 6.574 (s, 1H, Im), 7.374 (s, 1H, Im); 13.469, 13.726, 17.218, 17.594, 20.539, 21.180, 21.872, 20.047, 27.099, 27.575, 28.094, 29.044, 29.660, 29.780, 30.371, 30.804, 31.261, 33.383, 56.657, 120.951, 133.69, 134.577, 176.65.

$[P_{66614}]_2$[AA-Im]-CO_2: ^{1}H NMR (DMSO, ppm): 0.859 (t, 6H,CH_3), 0.879 (t, 18H, CH_3), 1.244 (m, 32H, CH_2), 1.286(m, 28H, CH_2), 1.379 (m, 16H, CH_2), 1.457 (m, 16H, CH_2), 1.567 (m, 4H, CH_2), 2.174 (m, 12H, CH_2), 2.676 (m, 1H, CH_2), 2.866 (m, 2H, NH_2), 3.539 (t, 2H, CH_2), 6.654 (s, 1H, Im), 7.419 (s, 1H, Im); ^{13}C NMR (DMSO, ppm): 13.860, 13.924, 17.344, 17.720, 20.651, 21.283, 21.893, 22.002, 22.180, 27.160, 27.622, 28.211, 28.761, 28.822, 29.078, 29.117, 29.772, 29.892, 29.996, 30.116, 30.207, 30.312, 30.492, 30.915, 31.394, 56.027, 121.379, 131.739, 133.828, 157.44, 174.701.

$[P_{66614}]_2$[AA-Ind]: ^{1}H NMR (DMSO, ppm): 0.858 (t, 6H,CH_3), 0.878 (t, 18H, CH_3), 1.244 (m, 32H, CH_2), 1.289 (m, 28H, CH_2), 1.376 (m, 16H, CH_2), 1.453 (m, 16H, CH_2), 1.566 (m, 4H, CH_2), 2.182(m, 12H, CH_2), 2.483 (m, 1H, CH), 3.060 (m, 2H, CH_2), 6.921(t, 1H, Ph), 7.014(t, 1H, Ph), 7.099(s, 1H, Im), 7.297(d, 1H, Ph), 7.508(d,1H, Ph); ^{13}C NMR (DMSO, ppm): 13.455, 13.871, 17.282, 17.659, 21.600, 21.264, 21.858, 21.960, 22.138, 27.138, 27.612, 28.153, 28.806, 29.017, 29.060, 29.738, 29.585, 29.952, 30.151, 30.455, 30.865, 31.342, 32.802, 57.170, 117.258, 113.252,

117.609, 118.492, 120.273, 123.095, 127.834, 136.397, 176.783.

$[P_{66614}]_2$[AA-Ind]-CO_2: ^{1}H NMR (DMSO, ppm): 0.860 (t, 6H, CH_3), 0.880 (t, 18H, CH_3), 1.245 (m, 32H, CH_2), 1.286(m, 28H, CH_2), 1.378 (m, 16H, CH_2), 1.454 (m, 16H, CH_2), 1.567 (m, 4H, CH_2), 2.177(m, 12H, CH_2), 2.897 (m, 1H, CH), 3.122 (m, 2H, CH_2), 6.933(t, 1H, Ph), 7.030(t, 1H, Ph), 7.107 (s, 1H, Im), 7.308 (d, 1H, Ph), 7.494(d,1H, Ph); ^{13}C NMR (DMSO, ppm): 13.451, 13.878, 13.947, 17.321, 17.697, 20.608, 20.643, 21.198, 21.290, 21.903, 22.014, 22.194, 27.128, 27.614, 28.22, 28.770, 28.833, 29.128, 29.165, 29.185, 29.769, 29.889, 29.992, 30.112, 30.212, 30.317, 30.501, 30.924, 31.405, 31.317, 56.936, 111.350, 117.748, 118.380, 121.274, 123.606, 127.902, 136.157, 165.622, 174.403.

$[P_{66614}]_2$[AA-Su]: ^{1}H NMR (DMSO, ppm): 0.850 (t, 6H, CH_3), 0.872 (t, 18H, CH_3), 1.236 (m, 32H, CH_2), 1.281 (m, 28H, CH_2), 1.378 (m, 16H, CH_2), 1.462 (m, 16H, CH_2), 1.557 (m, 4H, CH_2), 1.829 (m, 1H, CH), 2.417 (m, 2H, CH_2), 2.703 (t, 2H, CH_2); ^{13}C NMR (DMSO, ppm): 13.705, 17.289, 17.665, 20.599, 21.811, 22.080, 28.186, 28.740, 29.036, 29.080, 29.718, 29.839, 29.970, 30.090, 30.148, 30.253, 30.433, 31.309, 32.233, 49.614, 55.911, 176.331.

$[P_{66614}]_2$[AA-Su]-CO_2: ^{1}H NMR (DMSO, ppm): 0.840 (t, 6H, CH_3), 0.862 (t, 18H, CH_3), 1.226 (m, 32H, CH_2), 1.275 (m, 28H, CH_2), 1.378 (m, 16H, CH_2), 1.460 (m, 16H, CH_2), 1.558 (m, 4H, CH_2), 1.811 (m, 1H, CH), 1.950 (m, 2H, CH_2), 2.484 (t, 2H, CH_2); ^{13}C NMR (DMSO, ppm): 13.731, 17.386, 17.762, 20.677,21.267, 21.875, 21.989, 22.163, 27.124, 27.637, 28.277, 28.839, 29.133, 29.185, 29.758, 29.878, 30.015, 30.134, 30.235, 30.342, 30.502, 30.931, 31.403, 48.653, 55.368, 60.622, 157.454, 158.358, 174.219.

$[P_{66614}]_2$[Am-iPA]: ^{1}H NMR (DMSO, ppm): 0.860 (t, 6H, CH_3), 0.878 (t, 18H, CH_3), 1.247 (m, 32H, CH_2), 1.283 (m, 28H, CH_2), 1.374 (m, 16H, CH_2), 1.454(m, 16H, CH_2), 2.193 (m,16H, CH_2), 4.390 (s, 2H, NH_2), 6.997 (s, 2H, Ph), 7.528 (s 1H, Ph); ^{13}C NMR (DMSO, ppm): 14.261, 17.851, 18.228, 21.146, 22.332, 22.608, 28.708, 29.557, 29.602, 30.225, 30.346, 30.463, 30.582, 30.967, 56.237, 116.211, 122.194, 141.672, 145.995, 170.326.

$[P_{66614}]_2$[Am-iPA]-CO_2: ^{1}H NMR (DMSO, ppm): 0.857 (t, 6H, CH_3), 0.869 (t, 18H, CH_3), 1.244 (m, 32H, CH_2), 1.279 (m, 28H, CH_2), 1.368 (m, 16H, CH_2), 1.458(m, 16H, CH_2), 2.188 (m, 16H, CH_2), 7.092 (s, 2H, Ph), 7.604 (s 1H, Ph); ^{13}C NMR(DMSO, ppm): 14.213, 17.900, 18.276, 21.174, 22.338, 22.628, 28.739, 29.298, 29.642, 30.230, 30.350, 30.473, 30.599, 30.982, 56.278, 116.382, 119.946, 139.760, 146.761, 156.544, 157.669, 170.883.

4. References

1. X. Y. Luo, X. Y. Lv, G. L. Shi, Q. Meng, H. R. Li and C. M. Wang, *Aiche J.*, 2019, **65**, 230-238.
2. F. F. Chen, K. Huang, Y. Zhou, Z. Q. Tian, X. Zhu, D. J. Tao, D. E. Jiang and S. Dai, *Angew. Chem., Int. Edit.*, 2016, **55**, 7166-7170.
3. Y. J. Huang, G. K. Cui, Y. L. Zhao, H. Y. Wang, Z. Y. Li, S. Dai and J. J. Wang, *Angew. Chem., Int. Edit.*, 2017, **56**, 13293-13297.
4. Y. Huang, G. Cui, H. Wang, Z. Li and J. Wang, *J. CO_2 Util.*, 2018, **28**, 299-305.
5. X. Y. Luo, Y. Guo, F. Ding, H. Q. Zhao, G. K. Cui, H. R. Li and C. M. Wang, *Angew. Chem., Int. Edit.*, 2014, **53**, 7053-7057.
6. F. Ding, X. He, X. Y. Luo, W. J. Lin, K. H. Chen, H. R. Li and C. M. Wang, *Chem. Commun.*, 2014, **50**, 15041-15044.
7. X. Y. Luo, X. Y. Chen, R. X. Qiu, B. Y. Pei, Y. Wei, M. Hu, J. Q. Lin, J. Y. Zhang and G. G. Luo, *Dalton T.*, 2019, **48**, 2300-2307.
8. J. Guzmán, C. Ortega-Guevara, R. G. de León and R. Martínez-Palou, *Chem. Eng. Techn.*, 2017, **40**, 2339-2345.
9. M. Vafaeezadeh, J. Aboudi and M. M. Hashemi, *Rsc Adv.*, 2015, **5**, 58005-58009.
10. T. B. Lee, S. Oh, T. R. Gohndrone, O. Morales-Collazo, S. Seo, J. F. Brennecke and W. F. Schneider, *J. Phy. Chem. B*, 2016, **120**, 1509-1517.
11. S. Saravanamurugan, A. J. Kunov-Kruse, R. Fehrmann and A. Riisager, *Chemsuschem*, 2014, **7**, 897-902.
12. Y. Zhang, Z. Wu, S. Chen, P. Yu and Y. Luo, *Ind. Eng. Chem. Res.*, 2013, **52**, 6069-6075.
